# Microplastic contamination and removal efficiency in greywater treatment using a membrane bioreactor

**DOI:** 10.3389/fmicb.2025.1519230

**Published:** 2025-05-22

**Authors:** Suda Ittisupornrat, Chayanin Namyuang, Athit Phetrak, Paranee Sriromreun, Suthida Theepharaksapan

**Affiliations:** ^1^Department of Climate Change and Environment, Climate Change and Environmental Research Center, Pathumthani, Thailand; ^2^Department of Social and Environmental Medicine, Faculty of Tropical Medicine, Mahidol University, Bangkok, Thailand; ^3^Department of Chemical Engineering, Faculty of Engineering, Srinakharinwirot University, Nakhon Nayok, Thailand; ^4^Department of Civil and Environmental Engineering, Faculty of Engineering, Srinakharinwirot University, Nakhon Nayok, Thailand; ^5^Center of Excellence in Rail System Technology and Civil Engineering Material Innovation for Sustainable Infrastructure, Strategic Wisdom and Research Institute, Srinakharinwirot University, Bangkok, Thailand

**Keywords:** microplastic contamination, membrane bioreactor, greywater treatment, polyester fibers, bacterial community

## Abstract

Microplastic (MP) contamination in aquatic environments is a critical concern due to its potential effects on aquatic ecosystems. MP contamination is often unsatisfactorily eliminated using conventional wastewater treatment systems. Membrane bioreactor (MBR) is a modern solution for wastewater treatment offering significant advantages over traditional activated sludge systems, such as a smaller footprint and the ability to produce high-quality effluent. In this study, a pilot-scale MBR was conducted to evaluate MP removal from real greywater. The overall treatment performance for MP removal reached up to 90%, with the MP concentration in the permeate effluent being 0.02 MP L^−1^. The major MP size distribution was 101–300 μm, with polypropylene as the predominant MP type. Remarkably, polyester fibers were highly predominant in the suspended sludge. Furthermore, *Alphaproteobacteria*, *Bacteroidetes*, and *Actinobacteria* were the predominant communities in the MBR sludge, which preferably formed a biofilm associated with MP accumulation. This study underscores the potential of MBR technology for efficient MP removal in household buildings, contributing to the mitigation of MP discharge into the environment. Implementing MBR systems is a crucial step toward safeguarding aquatic ecosystems and preserving environmental integrity with respect to the corresponding increase in MP pollution.

## 1 Introduction

Managing waste, especially plastic waste, presents a significant challenge for many countries. Developing effective waste management strategies is essential, particularly for highly durable plastics that degrade very slowly in the environment. Over time, the extensive production and use of plastics have led to widespread pollution. Microplastics (MPs), plastic particles < 5 mm (Hongprasith et al., 2020), originate from various sources, both primary and secondary MPs. Sources of MPs include the direct manufacture of tiny plastic pellets and microbeads, frequently employed as constituents in cosmetics, synthetic textiles, and personal care products such as exfoliating scrubs, toothpaste, and lotions. Additionally, MPs can result from the breakdown of larger plastic items. These plastics are smaller fragments that persist in the environment, causing pollution (Hidalgo-Ruz et al., 2012). Thus, MPs are now pervasive in the environment, accumulating in food chains and posing toxicity risks (Teuten et al., 2009; Setälä et al., 2014). Their high surface area allows them to adsorb various environmental pollutants, including heavy metals and persistent organic pollutants. This ability to accumulate contaminants raises concerns about bioaccumulation and possible risks for ecosystems and human health (Rodrigues et al., 2019).

Research on the prevalence of MP pollution in aquatic environments has drawn considerable global attention. Numerous studies have reported the widespread presence of MPs in natural water sources worldwide (Kunz et al., 2023; Mennekes and Nowack, 2023). Understanding the pathways by which MPs move through these environments is crucial. Once discharged into water bodies, MPs can accumulate within wastewater treatment systems (Talvitie et al., 2017; Lares et al., 2018; Kittipongvises et al., 2022). Wastewater treatment systems are significant sources of secondary MP contamination (Habib et al., 2020; Liu et al., 2021).

Greywater, defined as domestic water excluding toilet waste, typically accounts for 50–80% of the total household water usage (Santasmasas et al., 2013) and is a major contributor to MP contamination. MPs from personal care products, laundry effluents, and cleaning agents enter the wastewater stream, eventually contaminating wastewater treatment systems (Esfandiari and Mowla, 2021; Jessieleena et al., 2023). Among these, synthetic fibers released during laundering are a predominant type of MPs found in greywater, primarily comprising polyester (PES), polyamide (PA), polypropylene (PP), and acrylic, with PES and PA accounting for 14–50% of the total MPs (Pedrotti et al., 2021).

Although some studies have examined the MP removal efficiency in greywater using methods coagulation combined with dissolved air flotation, achieving up to 90% removal (Esfandiari and Mowla, 2021), these were conducted at a laboratory scale with synthetic greywater and specific MP polymers such as polyethylene (PE) and microfiber. This may not fully reflect real greywater contamination and treatment conditions. Furthermore, limited studies have been conducted on MP removal in membrane bioreactors (MBRs) for MP removal in real greywater treatment. Existing research mainly compares the performance of MBRs to that of conventional activated sludge (CAS) processes in municipal wastewater treatment plants. For instance, Lares et al. (2018) reported that MBRs performed well in MP removal, with MBR permeate containing 0.4 MP L^−1^, compared to 1.0 MP L^−1^ in the final effluent of the CAS process, attributed to the microfiltration process. Similarly, Bayo et al. (2020) found that MBRs achieved better MP removal than rapid sand filtration in urban wastewater treatment. Recent studies further support these findings, demonstrating that MBR systems combined with ultrafiltration (UF) and reverse osmosis (RO) can achieve MP removal efficiencies of up to 98% (Cai et al., 2022). Additionally, Talvitie et al. (2017) reported that MBRs reduced MP concentrations from 6.9 ± 1.0 MP L^−1^ in the influent to as low as 0.005 ± 0.004 MP L^−1^ in the treated effluent, highlighting their superior efficiency over conventional treatment methods. These findings underscore the potential of MBR technology in reducing MP discharge into the environment while maintaining high-quality effluent. Additionally, MBRs require a smaller physical footprint compared to conventional treatment processes, making them suitable for urban and space-limited applications. Due to the membrane filtration step, MBR-treated effluent has significantly lower turbidity and suspended solids compared to conventional systems, ensuring higher water quality for potential reuse. Furthermore, MBRs perform efficiently under low organic-loading conditions, making them advantageous for decentralized and household greywater treatment. However, MBR performance specifically for treating MPs in real greywater remains unexplored. Therefore, identifying the types and loads of MPs in real greywater treated by MBR is essential for understanding their fate and stabilizing the performance of MBR treatment.

This study employed a pilot-scale MBR to treat real greywater from a dormitory. The research aimed to evaluate the performance of the MBR in removing MP while quantifying and characterizing MP contaminants in the permeate effluent and the sludge. This study also examined the predominant bacterial communities in the MBR sludge that could be associated with MP accumulation. Identifying the dominant MP types provides valuable insights into improving the efficiency of the MBR in MP removal from real greywater and classifying sources of contamination, supporting the development of targeted waste management strategies.

## 2 Materials and methods

### 2.1 MBR operation

**Figure 1:**
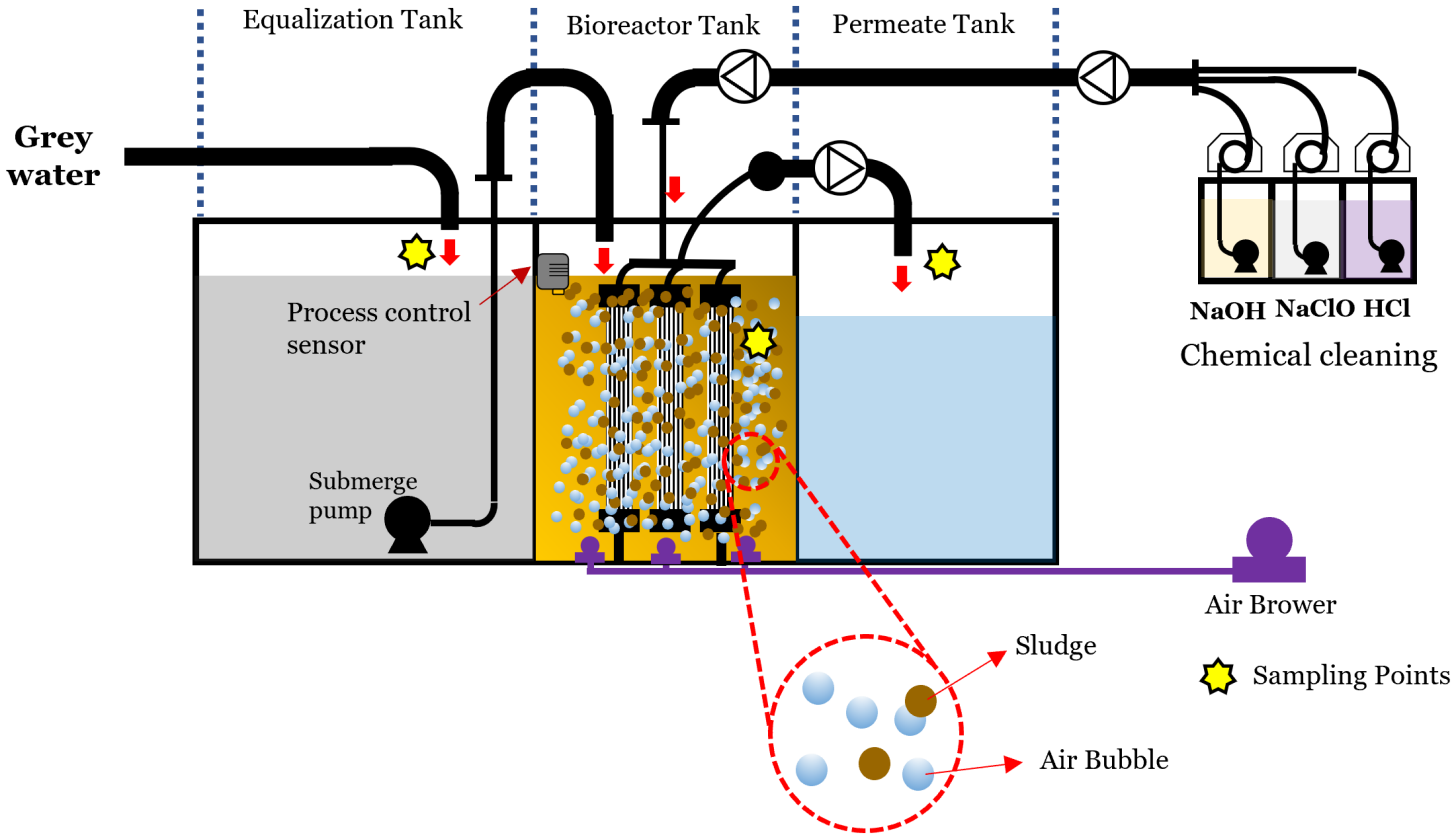
Schematic diagram of MBR pilot plant showing the different compartments, flow directions, and main instruments and equipment.

Figure 1 illustrates the schematic diagram of the MBR system. This pilot plant comprised several key components: a membrane chamber, an aerobic tank with a capacity of 5 m^3^, a permeate tank, and multiple pumps. Submerged hollow fiber microfiltration membranes with a nominal pore size of 0.1 μm (provided by Sumitomo, Japan) covered a total surface area of 36 m^2^ within the membrane chamber. Aeration was employed to clean the membrane surface. An electrical controller linked to a level sensor connected to the feed pump maintained a consistent water level in the aerobic tank. The MBR operated to process a maximum of 10 m^3^ of wastewater daily, with a continuous hydraulic retention time of 12 h and no sludge withdrawal for 5 years. Excess sludge from the CAS process served as seed sludge and was introduced into the aeration tank. The operational cycle comprised a filtration time of 9 min and a relaxation time of 1 min. Throughout the experimental period, the average permeate flux was consistently regulated at 11.5 L m^−2^ h^−1^.

The system was installed to treat greywater from a dormitory located in a university residential zone in Nakhon Nayok Province, Thailand. The eight-story building consists of 64 rooms, including 48 single rooms and 16 family rooms. Its sanitary system separates blackwater from toilets to a septic tank, while greywater from showers, hand-wash basins, kitchens, and laundry is directed to the treatment system. Sampling was conducted during February–March 2023, during which the greywater influent was continuously fed into the MBR system.

### 2.2 Sampling and collection of water and sludge samples

All samples were collected at two time points and were referred to as I and II. The second sampling was conducted one month after the first. After passing through a 1 cm screen chamber, influent samples with a volume of 1,000 L were collected. These samples were transferred to a series of four stainless steel test sieves with mesh sizes of 5 mm, 1 mm, 300 μm, and 100 μm using a submerged pump (Razeghi et al., 2021). Particles retained by the 5 mm sieve were discarded, whereas those retained by the 1 mm, 300 μm, and 100 μm sieves were gently rinsed with distilled water and individually collected into glass bottles. Thorough rinsing with distilled water was conducted to ensure the proper transfer of all particles. Similarly, the permeate from the MBR pilot plant was collected using the same method. Activated sludge samples (1 L) were gathered using a 10 L stainless steel bucket and transferred into glass bottles. All samples were transported to the laboratory and stored at 4°C in the dark until further processing.

### 2.3 Examination and identification of MPs

The water and sludge samples were treated based on the method proposed by Masura et al. (2015), with minor adjustments. The sieved samples were placed in a 60°C drying oven overnight until almost dry. The pretreated samples underwent digestion via wet peroxide oxidation to remove organic matter contamination. This oxidation process involved the addition of 20 mL of a 0.05 M aqueous Fe (II) solution and 20 mL of 30% hydrogen peroxide (H_2_O_2_) to the pretreated samples. The mixture was allowed to settle for 5 min at room temperature before agitation on a hotplate with a magnetic stirrer at 60°C, covered with a watch glass. Depending on the organic matter content in the sample, an additional 20 mL of H_2_O_2_ might have been added to complete the oxidative reaction. Density separation was conducted using a saturated sodium chloride solution. After the samples were left to settle overnight, the settled particles were discarded. The supernatant was then filtered through an Anodisc filter of 0.2 μm. The filtered samples were kept in glass Petri dishes before further analysis.

To identify the samples, optical microscopy and Fourier transform infrared microscopy (ALPHA II, Bruker Optik GmbH, Germany) were utilized. The analysis was conducted in the transmittance mode, with 128 scans taken to generate spectra ranging from 1,200 to 4,000 cm^−1^ at a spectral resolution of 4 cm^−1^. These spectra were compared to the libraries provided by Bruker for identification.

### 2.4 Quality assurance and quality control

To prevent MP contamination, cotton clothing and gloves were worn during the experiments, and only nonplastic equipment was used. All glassware and stainless-steel equipment were washed with detergent and rinsed with ultrapure (Wesch et al., 2017). For blank controls, ultrapure water was filtered using the same sieve sets for sample collection.

### 2.5 Water quality analysis

Dissolved oxygen (DO), pH, and temperature were measured using appropriate probes (YSI 60, United States). The chemical oxygen demand (COD) was analyzed using Hach methods (HACH, United States). Total nitrogen (TN) was analyzed by the total organic carbon analyzer (TOC-L, Shimadzu, Japan). Turbidity was determined using a turbidity meter (Thermo Fisher, United States). Total suspended solids (TSS) were analyzed using the standard method (APHA, 2017). The mixed liquor suspended solids (MLSS) concentration was determined using Japanese standard methods (JSWA, 1997).

### 2.6 DNA extraction, sequencing, and bioinformatic processing analysis

DNA extraction and sequencing were conducted by the Zymobiomics service (Zymo Research, Irvine, CA, United States). DNA extraction was performed using the ZymoBIOMICs-96 MagBead DNA Kit. Zymo Research custom-designed two primer sets, V1-V2 and V3-V4, to ensure optimal coverage while maintaining high sensitivity. The PCR reactions were conducted in real-time PCR machines to control cycles and minimize PCR chimera formation. The final library was sequenced on an Illumina^®^ MiSeq™ platform with a v3 reagent kit (600 cycles), with the addition of a 10% PhiX spike-in. Unique amplicon sequences were identified from the raw reads through analysis using the Dada2 pipeline (Callahan et al., 2018).

Chimeric sequences were also removed with the Dada2 pipeline. Taxonomic classification was conducted using Uclust from Qiime version 1.9.1 (Caporaso et al., 2010), using the Zymo Research Database. The significant abundance among different groups was identified by LEfSe (Segata et al., 2011). The relative bacterial abundance was characterized at the phylum level, and a phylum with <2% an abundance was considered a minor phylum. Class and order were also categorized together, and percentage levels of the identified genus are shown.

## 3 Results and discussion

### 3.1 Operating conditions of the MBR for treating greywater

The MBR operated at a capacity of 10.2 ± 1.1 m^3^ d^−1^. Table 1 details the water quality characteristics of the greywater and MBR effluent and the overall treatment performance. Throughout the operation, the pH of the influent and MBR effluent remained neutral at ambient temperature. DO levels in the influent were <0.1 mg L^−1^, whereas the DO levels in the effluent increased to 4.6 mg L^−1^ due to aeration in the MBR tank. The influent exhibited average COD and TN levels of 93.5 mg L^−1^ and 8.8 mg L^−1^, respectively. Conversely, the MBR effluent showed average COD and TN levels of 24.2 mg L^−1^ and 4.0 mg L^−1^, respectively, with average treatment efficiencies of 74 and 55%, respectively. Greywater composition primarily originates from bath, shower, and laundry sources, resulting in low COD and TN concentrations. When comparing the treatment performance of greywater between low and high loading capacity, it was found that the removal efficiency of the MBR process could be a stable treatment (Ittisupornrat and Theepharaksapan, 2023). Due to the low COD concentration in the influent (ranging from 57 to 130 mg L^−1^), the average MLSS concentration increased from 0.8 to 1.4 g L^−1^ after one month of operation, reaching an average of 1.1 g L^−1^. In addition, various anti-bacterial agents and some surfactants from shampoos and body soaps may contribute to inhibit bacterial growth (Liu et al., 2005; Lamine et al., 2012). The average TSS level in the influent was 9.4 mg L^−1^, corresponding to the turbidity value of 16.1 NTU. Meanwhile, TSS and turbidity were completely removed through the treatment, with values higher than 99% due to microfiltration by membrane (Ittisupornrat et al., 2019).

**Table 1:** Characteristic of water quality of greywater and MBR effluent.

Parameter	Unit	Influent	MBR effluent	Removal efficiency (%)
pH	–	7.0	7.5	–
DO	mg L^−1^	0.1	4.6	–
COD	mg L^−1^	93.5	24.2	74
TN	mg L^−1^	8.8	4.0	55
Turbidity	NTU	16.1	0.3	>99
TSS	mg L^−1^	9.4	<0.2	>99

The results reported here are given as mean (*n* = 2).

### 3.2 Removal efficiency and characteristics of MPs in the MBR

**Figure 2:**
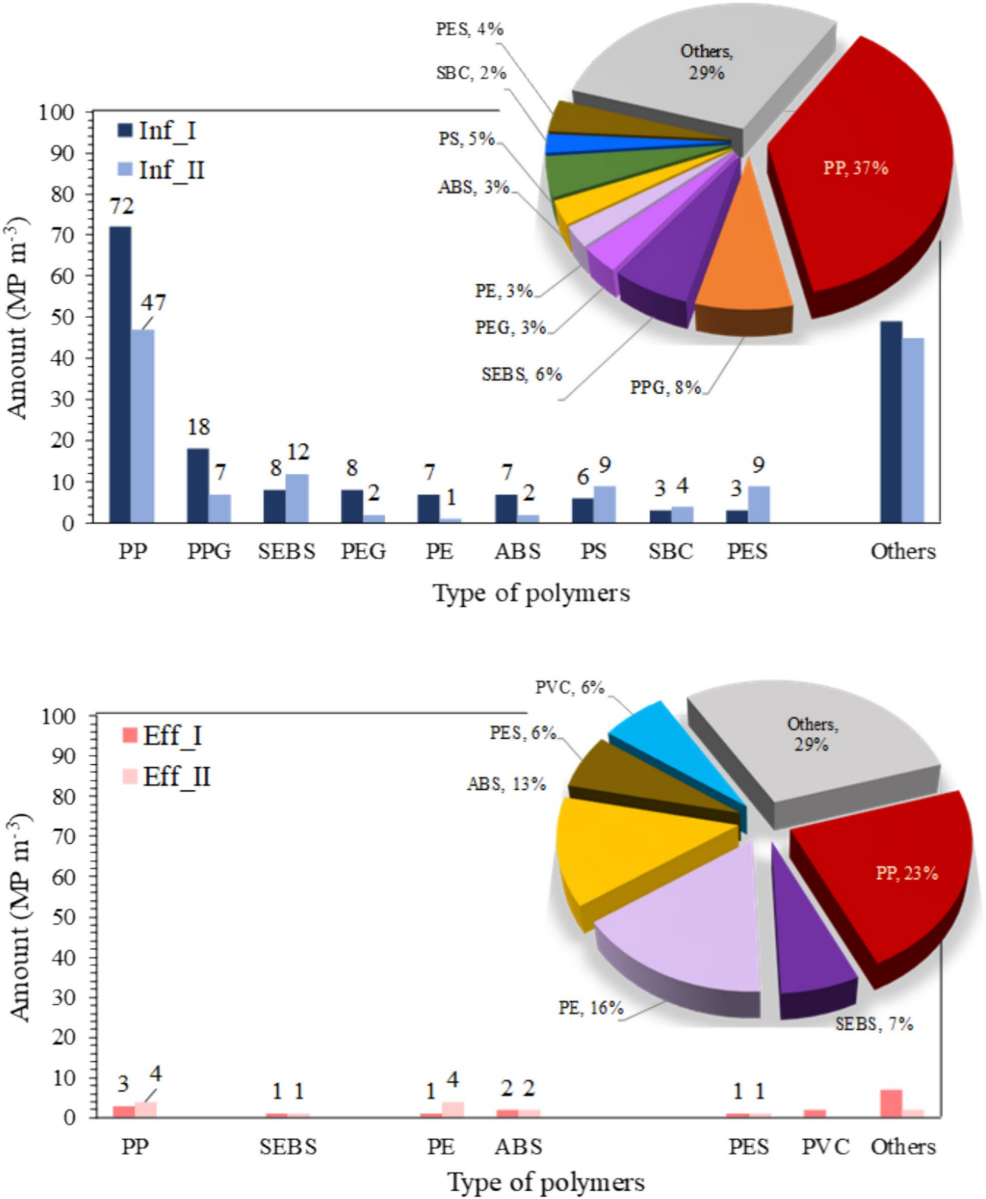
The predominant polymer types in microplastic contamination were analyzed in the influent (top) and effluent (bottom) samples, with corresponding percentages shown in the pie charts. I and II represent the first and second water sampling events, respectively. PP, polypropylene; PPG, polypropylene glycol; SEBS, styrene–ethylene–butylene-styrene; PEG, polyethylene glycol; PE, polyethylene; ABS, acrylonitrile butadiene styrene; PS, polystyrene; SBC, copolymer styrene–butadiene; PES, polyester; PVC, polyvinylchloride.

Figure 2 presents the analysis of MP contamination and highlights the predominant polymer types in the influent and effluent samples across two sampling events. The list of influents includes PP, polypropylene glycol (PPG), styrene–ethylene–butylene–styrene (SEBS), polyethylene glycol (PEG), PE, acrylonitrile butadiene styrene (ABS), polystyrene (PS), copolymer styrene–butadiene (SBC), PES, and others, whereas the list of effluents includes PP, SEBS, PE, ABS, PES, polyvinyl chloride (PVC), and others. In the influent, PP was the most abundant polymer with concentrations ranging from 47 to 72 MP m^−3^, accounting for an average of 37% of MPs, followed by PPG (7–18 MP m^−3^) and SEBS (8–12 MP m^−3^). In the effluent, PP remained the dominant polymer with concentrations ranging from 3 to 4 MP m^−3^, representing 23% of MPs followed by PE (2 MP m^−3^) and ABS (1–2 MP m^−3^). This is in agreement with Wei et al. (2020), who reported that PP was the most prevalent polymer type in rural domestic wastewater. In addition to this polymer, PE, PS, and PES are commonly used in personal care products and cosmetics, and cleaning products. These primary and secondary MPs are derived from our everyday activities and are generated through abrasion of various packaging materials and synthetic clothing (Mishra et al., 2022; Jessieleena et al., 2023; Song and Li, 2023).

SEBS and ABS are technical thermoplastics widely used in typical applications, such as toys, automotive applications, general purpose molded goods, window and door seals, and electrical and electronic spare parts (Peydro et al., 2013). The proportion of different polymers varied only slightly between I and II when MPs from both samples were considered. PPG and PEG were the important plasticizers in the detergents, which were detected in influent samples but not in effluent samples (Piorkowska et al., 2006). This could be because these polymers are easily degraded by microorganisms (Kadac-Czapska et al., 2023).

**Figure 3:**
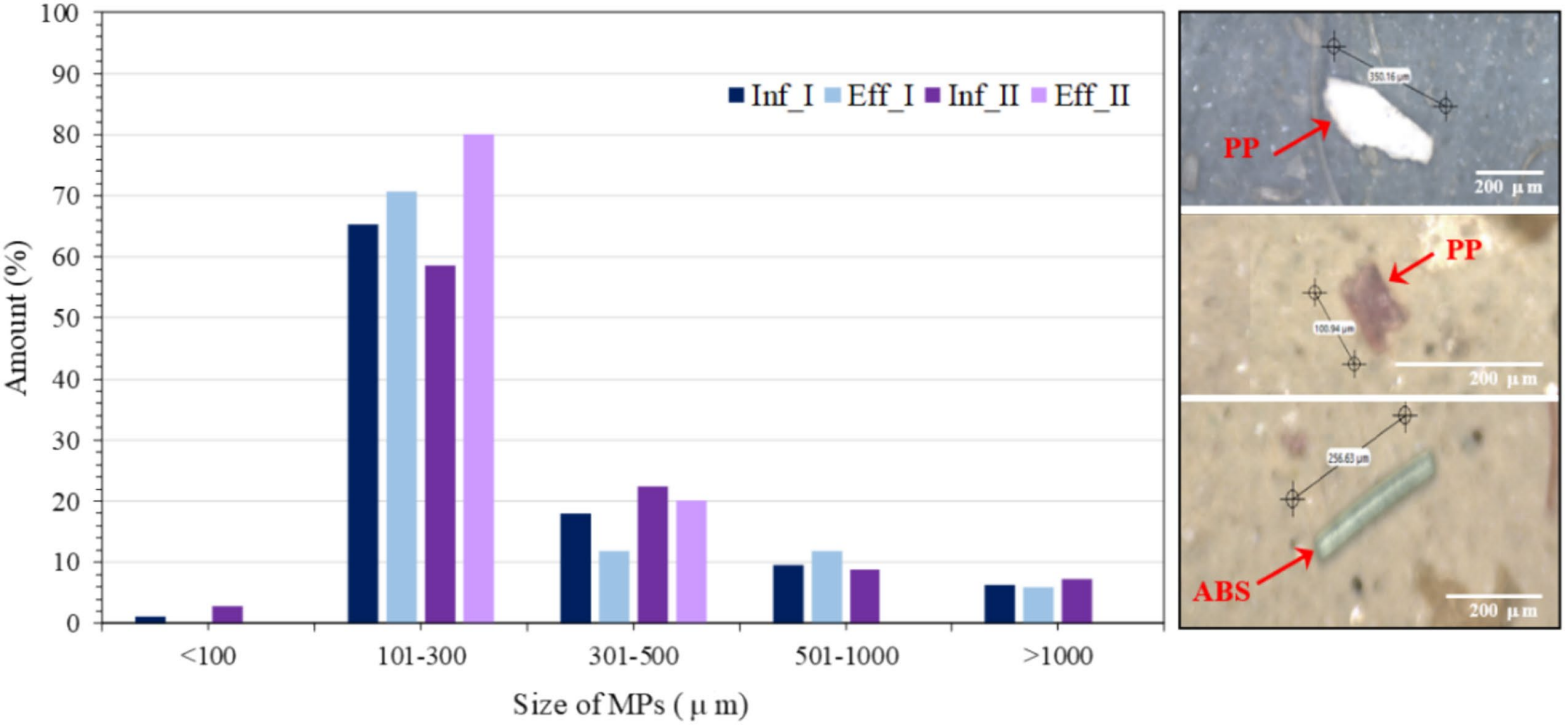
Size distribution and occurrence percentage of microplastics in the influent and MBR effluent. The right panel shows examples of microplastics. I and II represent the first and second water sampling events, respectively.

Figure 3 presents the MP size distribution and occurrence percentage in the influent and MBR effluent samples. Visual examples provided in Figure 3 (right) illustrate various MPs in the influent and effluent, including fragments and fibers. The MP size distribution is an important parameter associated with their removal efficiencies. In this study, the predominant size of MPs was 101–300 μm (only in length measurement) in both influent and MBR effluent, accounting for 62 and 76%, respectively, on average, followed by an MP size of 301–500 μm accounting for 20 and 16%, respectively. Meanwhile, MP sizes of 501–1,000 and > 1,000 μm were rarely measured (<10%), in accordance with (Egea-Corbacho et al., 2023), who described small-sized particles (100–355 μm) as the major size in influent and effluent samples. Similarly, Liu et al. (2019) found 65–87% of MPs < 1 mm in the influent originating from municipal wastewater but 81–91% in the effluent of the wastewater treatment process, implying that the origin MP particles are decomposed to secondary MPs by physical, chemical or biological processes during the operation (Song and Li, 2023). Furthermore, this experiment measured MPs only in the length mode (not thickness), and the observed MP size mostly seemed bigger than the nominal pore size of the membrane (0.1 μm). Based on the phenomena of membrane microfiltration, the pore size of the membrane should not allow such large particles to pass through, but it was observed in an effluent, perhaps due to leakage from long-term operation (Egea-Corbacho et al., 2023). Conversely, the high suction pressure used in membrane filtration may force small-sized MPs to pass through membrane pores. This observation aligns with previous studies reporting the presence of MPs in MBR effluents, indicating the potential risk of MP leakage from such systems (Talvitie et al., 2017; Bayo et al., 2020; Cai et al., 2022) effluent that there is a risk of MP release from MBR systems. However, the concentration of MPs released from the MBR was lower than that from the conventional wastewater treatment plant (Lares et al., 2018).

The average MPs found in the influent and effluent were 159.5 and 15.5 MP m^−3^, respectively, resulting in a 90.2% removal efficiency of MPs (Table 2). Results were in accordance with previous studies conducted in MBR, indicating that MP concentrations in the MBR permeate (0.4 MP L^−1^) were measured (Lares et al., 2018; Iyare et al., 2020), implying the system’s ability to efficiently reject large MP particles by membrane microfiltration (Li et al., 2020). The MBR process is strongly recommended as the best cost-saving technology for reducing MPs in the aquatic environment (Vuori and Ollikainen, 2022). During this operation, no membrane fouling was observed. However, membrane fouling is one of the main drawbacks of membrane separation. Several studies noted that different MP particle diameters influence the biofouling of MBRs in different ways (Wang et al., 2022; Acarer, 2023).

**Table 2:** Removal efficiency of microplastics in greywater by MBR.

No.	Influent	MBR effluent	Removal efficiency
	(MP m^−3^)	(MP m^−3^)	(%)
I	181	17	90.6
II	138	14	89.8
Average	159.5	15.5	90.2

I and II represent the first and second water sampling events, respectively.

### 3.3 Characteristics of MPs in the sludge

**Figure 4:**
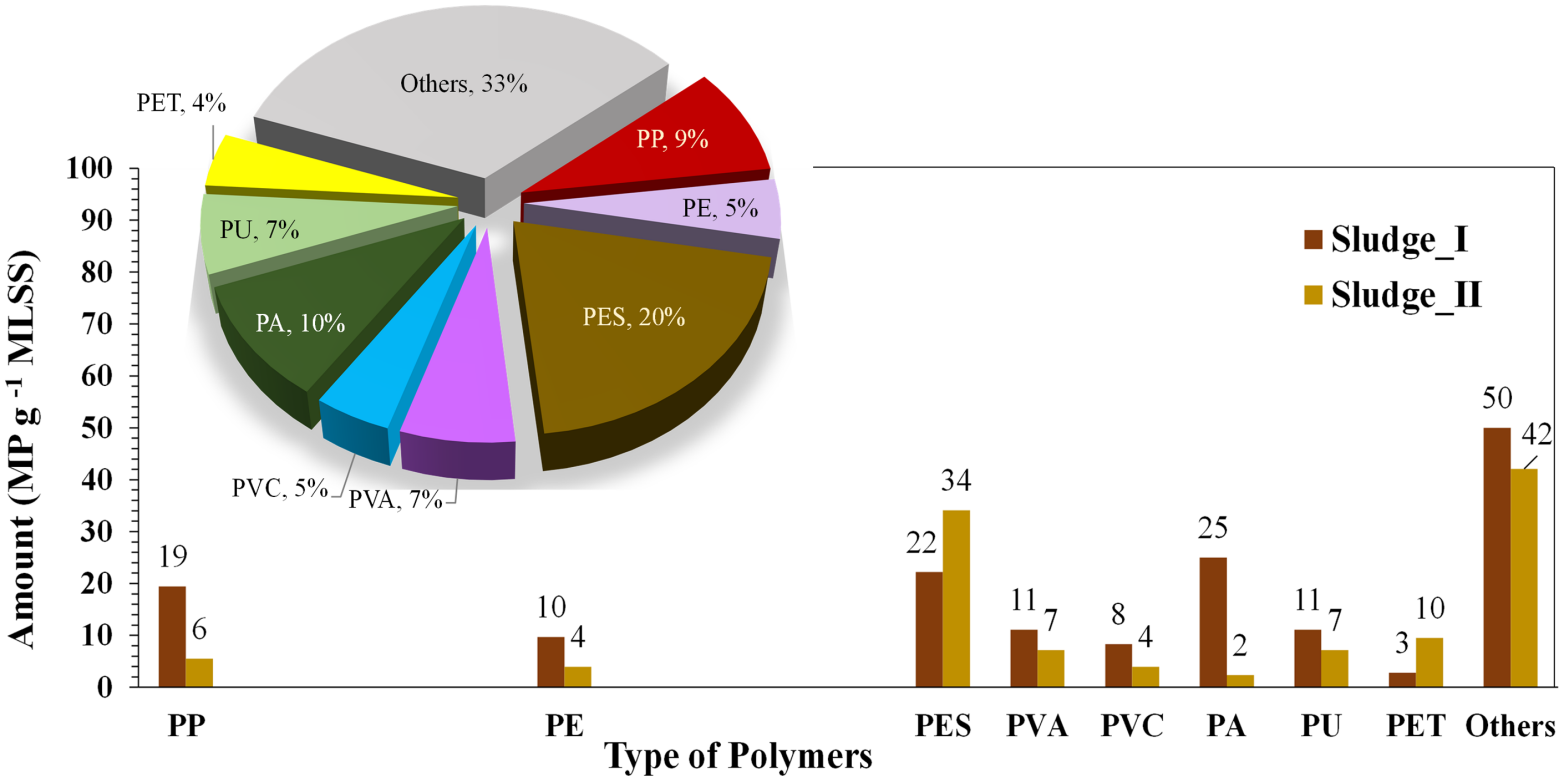
The predominant polymer types in microplastic contamination were analyzed in the sludge samples, with corresponding percentages shown in the pie charts. I and II represent the first and second sludge sampling events, respectively. Abbreviations: PP: polypropylene; PE: polyethylene; PES: polyester; PVA: polyvinyl alcohol; PVC: polyvinylchloride; PA: polyamide; PU: Polyurethane; PET: polyethylene terephthalate.

**Figure 5:**
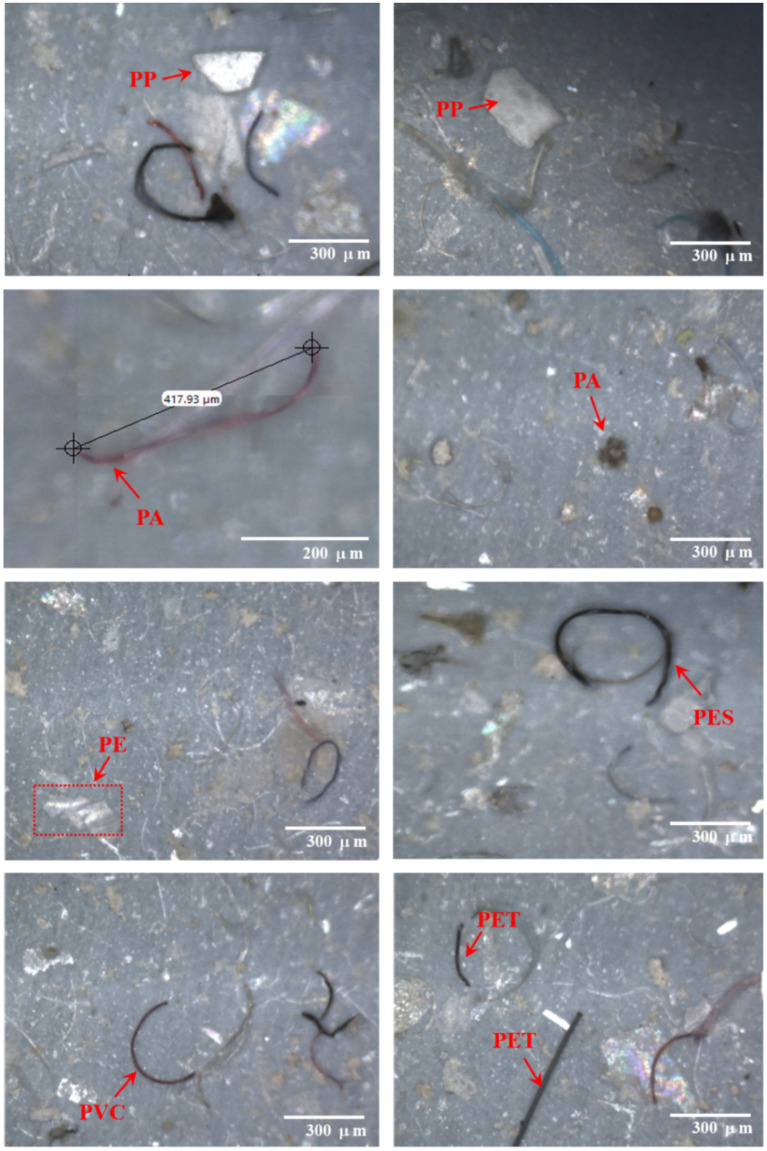
Occurrence of polymer types of microplastics in MBR sludge. PP, polypropylene; PA, polyamide; PE, polyethylene; PES, polyester; PVC, polyvinylchloride; PET, polyethylene terephthalate.

MPs in the MBR sludge were analyzed, revealing that PES was the major microfiber, with an average concentration of 28 MP g^−1^ MLSS, accounting for 20% of the total MPs (Figure 4; Table 3). PA and PP followed, with average concentrations of 14 and 13 MP g^−1^ MLSS, respectively. The accumulation characteristics of MPs in the sludge are presented in Figure 5. These results are in accordance with those of Lares et al. (2018), who found secondary MPs originating from microfibers (24.1 ± 6.1 MP g^−1^ dry weight) to be the dominant type of MPs in the MBR sludge. The observed PES may originate from washing of clothes (Hazlehurst et al., 2023). Among PP, PA, PU, PE, polyethylene terephthalate (PET), and PVC have also been widely used for producing clothes and textiles, as well as all common plastic materials used in daily life (Song and Li, 2023). Moreover, polyvinyl alcohol (PVA) was detected (7%), which is commercially used in laundry and dish detergent pods (Rolsky and Kelkar, 2021). Furthermore, some studies revealed that ≥90% of MPs removed in wastewater treatment plants are entrapped in the sludge (Reddy and Nair, 2022), and effective sludge management to mitigate their dispersion into the environment should be considered.

**Table 3:** Types of polymer in microplastics accumulation in sludge.

Sludge	Types of polymer in microplastics (MP g^−1^ MLSS)								
	PES	PP	PA	PVA	PU	PET	PE	PVC	Others
I	22	19	25	11	11	3	10	8	50
II	34	6	2	7	7	10	4	4	42
Average	28	13	14	9	9	6	7	6	46

I and II represent the first and second sludge sampling events, respectively. PES, polyester; PP, polypropylene; PA, polyamide; PVA, polyvinyl alcohol; PU, Polyurethane; PET, polyethylene terephthalate PE, polyethylene; PVC, polyvinylchloride.

### 3.4 Bacterial community in the MBR sludge

The bacterial community composition showed similarities between the first and second sampling events, with slight variations in relative abundance. The structure of the dominant phyla is shown in Supplementary Figure S1. The bacterial population was predominantly composed of *Proteobacteria* (40.5–50.7%), followed by *Actinobacteria* (10.1–11.5%) and *Bacteroidetes* (9.6–9.8%). *Proteobacteria* emerged as the most dominant phylum during MBR operation (Ittisupornrat et al., 2021) as they play a primary role in the biodegradation of various organic compounds within the MBR (Sanguanpak et al., 2019; Ittisupornrat and Theepharaksapan, 2023). Regarding MP concerns, although this study did not confirm the detection of the bacterial community on the MP surface, this study aligned with previous findings, indicating that *Proteobacteria* and *Bacteroidetes* are typically the predominant phyla detected in plastisphere environments (Gong et al., 2019; Yang et al., 2020; Aguila-Torres et al., 2022; Nguyen et al., 2022). In particular, Qiu et al. (2024) suggested that the main bacterial communities of *Proteobacteria*, *Actinobacteria*, and *Bacteroidetes* were involved in the metabolism of dissolved organic matter released by MP. Furthermore, previous research investigated their community biofilm on the surface membrane made from polyvinylidene fluoride polymers in treated greywater (Ittisupornrat and Theepharaksapan, 2023).

Although Wang et al. (2022) reported that the dominant PP could improve *Clostridia* abundance and reduce the *Proteobacteria* in MBR treating municipal wastewater, this study not obviously observed dominated *Clostridia* and declining *Proteobacteria*. In addition to the different origins of wastewater sources, the opposite results may be attributed to other intricate causes that might be further investigated. Some *Acidobacteria* (5.0–5.1%), *Chloroflexi* (4.4–5.6%), *Chlorobi* (0.6–2.0%), and *Nitrospirae* (1.6–2.1%) were slightly elevated. Only *Planctomycetes* was obviously decreased from 7.3 to 4.2%. The decreasing trend of this phylum was observed when MBR was simultaneously operated to treat greywater during a long-term period. However, the influence of MPs on this phylum remains unclear, presenting an opportunity for further research to understand its role and impact in MBR systems.

**Figure 6:**
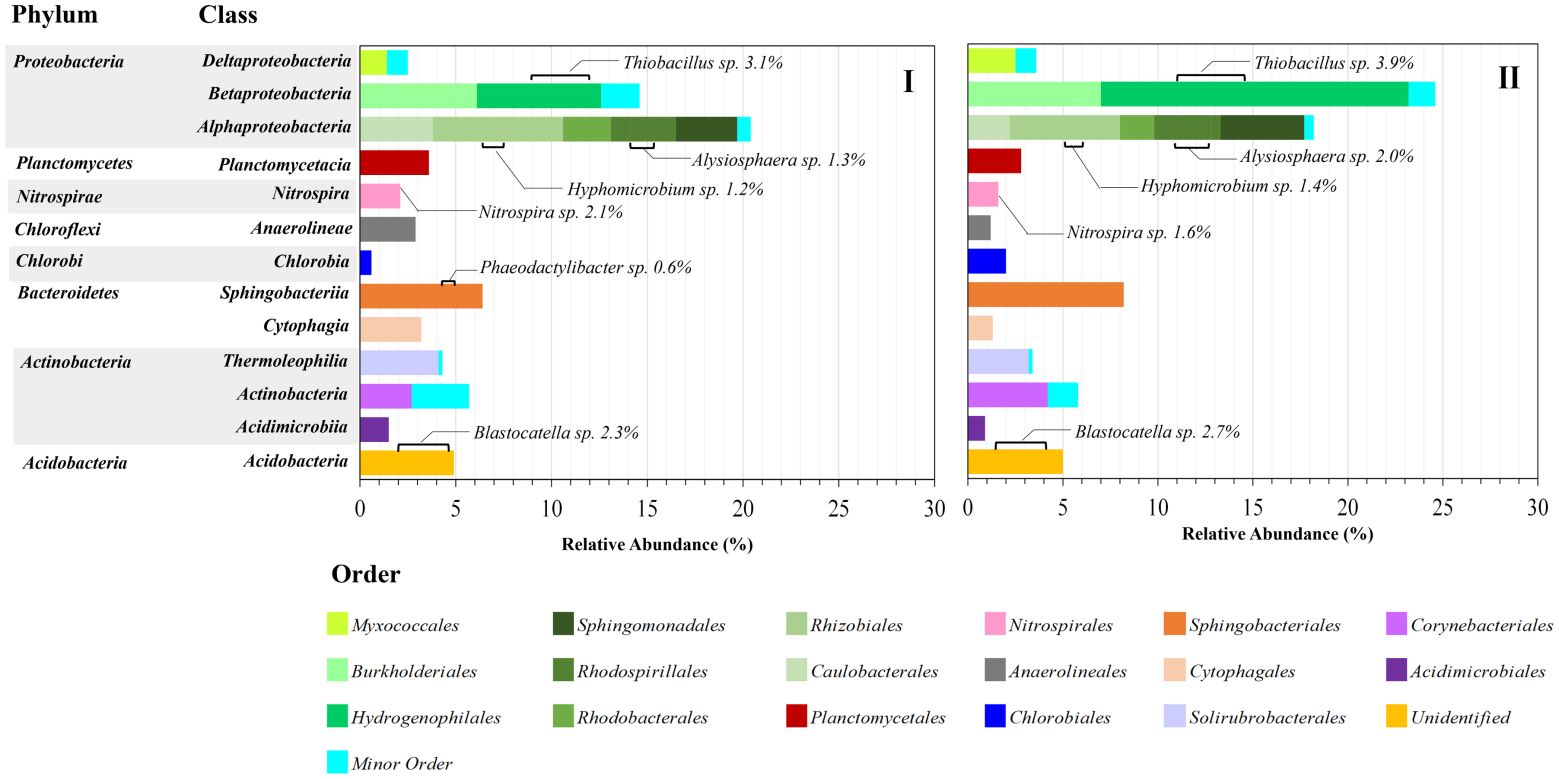
Relative abundance of the order level in each class and identified genus within each.

Figure 6 shows the distribution of the bacterial population at the order level in each class. *Alphaproteobacteria* (18.2–20.4%) and *Betaproteobacteria* (14.6–24.6%) were the most dominant, similar to Martínez-Campos et al. (2021), who reported that *Alphaproteobacteria* (24.2%) and *Betaproteobacteria* (21.4%) were dominantly observed. Within the *Alphaproteobacteria* class, *Caulobacterales, Rhizobiales, Rhodobacteriales, Rhodospirillales,* and *Shingomonadales* orders were observed, with *Rhizobiales* (5.8–6.8%) being the most predominant. Only two strains could be identified at the genus level: *Hyphomicrobium* sp. (1.2–1.4%) and *Alysiophaera* sp. (1.3–2.0%). These orders were observed to be predominant in the biofilm community (Ittisupornrat and Theepharaksapan, 2023). Many studies have pointed out that MPs may encourage bacterial adhesion and subsequently biofilm development and support biofilm community niches because MPs may act as a repository for bacteria (Yang et al., 2019; Martínez-Campos et al., 2021; Sooriyakumar et al., 2022; Ladeia Ramos et al., 2024). Similarly, Jiang et al. (2018) also mentioned that these orders represented important bacterial associations within bacterial communities of the plastisphere. Furthermore, many studies showed that MP biofilms may selectively harbor bacterial pathogens and antibiotic-resistant bacteria (Gong et al., 2019; Parsaeimehr et al., 2023). However, no pathogenic bacteria were found in the bacterial community population of this study, possibly because the greywater constituents contained various detergents and antibacterial agents that could inhibit bacterial pathogen generation. Within the *Betaproteobacteria* class, two orders of *Burkholderiales* (6.1–7.0%) and *Hydrogenophilales* (6.5–16.2%) were observed. *Hydrogenophilales* remarkably increased from 6.5 to 16.2%. Only one genus was identified as *Thiobacillus* sp. (3.1–3.9%), whereas the dominant genus (increased from 3.1 to 12.3%) could not be identified as belonging to this order. Regarding the *Acidobacteria* order, only *Blastocatella* sp. (2.3–2.7%) was identified. In addition, the abundance of other orders seemed relatively constant in I and II. Unfortunately, most bacteria could not be identified at the genus level. *Nitrospira* sp. (1.6–2.1%) was the main contributor to the nitrogen transformation that was observed in the *Nitrospirales* order resulting in high nitrogen removal performance, suggesting that the presence of MPs might not pose a threat to the abundance and functionality of nitrite-oxidizing bacteria (Cui et al., 2021; Wang et al., 2021). Similarly, Huang et al. revealed that MPs drive nitrification by enhancing the abundance of functional microorganisms in aquaculture pond waters, including ammonia- and nitrite-oxidizing bacteria (Huang et al., 2022).

Based on the acquired knowledge from this study, it suggests a possible link between microbial communities and MPs; the precise interactions and mechanisms remain to be fully elucidated. Further research is necessary to determine how these bacterial populations contribute to MP aggregation, degradation, or transport within the MBR system. To avoid any negative impact on MP contamination into the environment, Parsaeimehr et al. (2023) recommended the use of a closed and controlled wastewater treatment system. Therefore, MBR should be offered for prominent wastewater treatment processes to remove and retain MPs in the system (Vuori and Ollikainen, 2022).

### 3.5 Significant implications and future perspectives

Studies on the removal of MP using membrane technologies are ongoing. Lack of implemented standards or relevant policies for the detection, removal, or discharge of MPs is driving the research on applications of the membrane process focusing on MP removal.

This study highlights the significant potential of MBR technology in addressing the global challenge of MP pollution. The system achieved an average MP removal efficiency of 90%, reducing the effluent concentrations to ≤0.02 MP L^−1^, while achieving significant COD removal at 74%. These results underscore the ability of MBR to integrate microfiltration with biological activity, enabling simultaneous removal of MPs and decreasing COD. This dual functionality represents a novel advancement over conventional wastewater treatment methods, particularly for real greywater, where complex contaminants and low organic loads pose unique challenges. Furthermore, the role of dominant bacterial communities, such as *Alphaproteobacteria* and *Actinobacteria*, in biofilm formation enhances the efficiency of MP removal and offers insights into bacteria can aid in optimizing the MBR performance.

In addition to its technological benefits, MBR systems offer practical solutions for urban and residential wastewater treatment, where space and sustainability are critical. The compact design and high pollutant removal efficiency make MBR systems a promising alternative to conventional methods. Policymakers can integrate MBR technology into existing wastewater infrastructure to meet stricter environmental regulations, reduce MP discharge, and mitigate risks to aquatic ecosystems. Although MBR systems have higher initial costs than conventional methods, their long-term benefits are reduced environmental risks, lower maintenance needs for downstream ecosystems, and potential savings in regulatory compliance. A detailed cost–benefit analysis could further help in improving their economic feasibility, emphasizing their value as a sustainable investment in water management that should be more focused for further study. Furthermore, this study provides a foundation for future research into bacterial interactions with MPs, biofilm dynamics, and operational challenges such as membrane fouling and potential MP leakage. By focusing on these aspects, MBR systems can be further optimized to deliver enhanced performance and sustainability. Overall, these findings highlight the use of MBR technology for greywater treatment as a transformative solution for advancing wastewater treatment while protecting aquatic ecosystems from emerging pollutants.

## 4 Conclusion

Results of this study highlight the significant potential of MBR technology in mitigating MP pollution, achieving a removal efficiency of 90% and reducing the effluent MP concentrations to ≤0.02 MP L^−1^. These findings demonstrate that MBR systems can effectively address the growing environmental threat posed by MPs, offering a sustainable solution for greywater treatment in residential and urban applications. Furthermore, they underscore the role of bacterial communities, such as *Alphaproteobacteria* and *Actinobacteria*, in biofilm formation and enhanced MP removal. By integrating MBR technology into wastewater treatment frameworks, policymakers can reduce MP discharge into aquatic ecosystems and meet stricter environmental regulations. Future research should explore microbial interactions with MPs and address operational challenges to optimize MBR performance and sustainability. This study lays the foundation for advancing wastewater treatment technologies to protect ecosystems and improve environmental resilience.

## Data Availability

The original contributions presented in the study are publicly available. The sequence datasets of bacterial community for this study can be found in the NCBI Sequence Read Archive (SRA) under BioProject PRJNA1089773 (https://www.ncbi.nlm.nih.gov/bioproject/PRJNA1089773).

## Supplementary material

Supplementary Figure S1 Bacterial community compositions at the phylum level of the sludge samples in MBR. Abundance of phyla <2% was termed as Minor phyla. I and II represent the first and second sludge sampling events, respectively.
