# Supplementary figures and images for: Microplastic contamination and removal efficiency in greywater treatment using a membrane bioreactor
Source: Front Microbiol. 2025 May 22;16:1519230. doi: 10.3389/fmicb.2025.1519230 (PMC12137276; doi:10.3389/fmicb.2025.1519230)

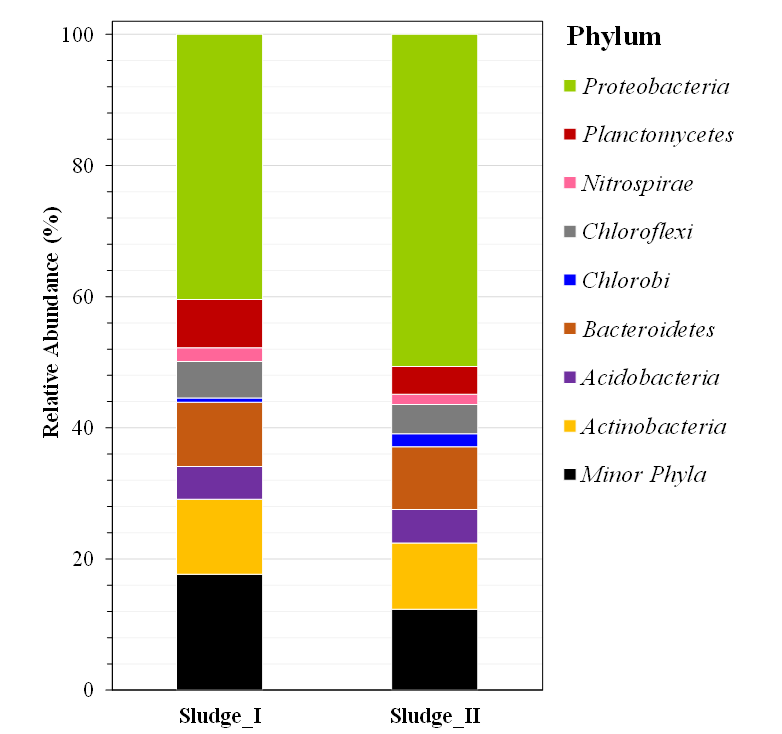

Supplement: Supplementary Figure S1 — Bacterial community compositions at the phylum level of the sludge samples in MBR. Abundance of phyla <2% was termed as Minor phyla. I and II represent the first and second sludge sampling events, respectively. [file Image_1.tif]
